# Detection of Central Compartment Lymph Node Metastasis of Thyroid Cancer: Usefulness of Intraoperative Thyroglobulin Measurement in Fine Needle Aspiration Washout with and Without Blue Dye Injection

**DOI:** 10.3390/cancers17030422

**Published:** 2025-01-27

**Authors:** Chiara Mura, Gian Luigi Canu, Giulia Lanzolla, Federico Cappellacci, Fabio Medas, Stefano Mariotti, Pietro Giorgio Calò, Francesco Boi

**Affiliations:** 1 Endocrinology Unit, Department of Medical Sciences and Public Health, University of Cagliari, 09100 Cagliari, Italy; chiara.mura@unica.it (C.M.); mariottistefano48@gmail.com (S.M.); francesco.boi@unica.it (F.B.); 2 Surgery Unit, Department of Surgical Sciences, University of Cagliari, 09100 Cagliari, Italy; gianl.canu@unica.it (G.L.C.); f.cappellacci@aoucagliari.it (F.C.); fabio.medas@unica.it (F.M.); pgcalo@unica.it (P.G.C.)

**Keywords:** thyroglobulin, intraoperative Tg-FNA, blue dye, central neck lymph nodes

## Abstract

**Background/Objectives**: The management of lymph node metastases of the central neck compartment (CNC) in differentiated thyroid carcinoma is debated. The intraoperative measurement of thyroglobulin (Tg) has gained attention in accurately detecting metastases, reducing unnecessary CNC dissections. **Methods**: A total of 37 patients underwent surgery. An intraoperative assay of thyroglobulin from fine needle aspiration (Tg-FNA) was performed on CNC lymph nodes, identified by blue dye injection in 15 patients (23 nodes, group A) and by palpation in 22 patients (35 nodes, group B). The Tg-FNA values were compared with histology to calculate the diagnostic accuracy. **Results**: In group A, the blue dye diffused widely, complicating lymph node identification: 2 were metastatic and 21 non-metastatic, with median Tg-FNA levels of 6236 ng/mL and 99.20 ng/mL, respectively. In group B, 8 were metastatic and 27 benign, with median Tg-FNA levels of 4063 ng/mL and 121 ng/mL (*p* < 0.0001), respectively. ROC analysis identified 500 ng/mL as a cutoff, achieving 100% sensitivity and 74% specificity in group B and 90% sensitivity and 70% specificity overall. Finally, among the non-metastatic lymph nodes, group A exhibited some cases of very high Tg-FNA values compared to group B, with lower accuracy for the cutoff, suggesting that colorant injection might lead to increased Tg-FNA levels. **Conclusions:** Blue dye injection showed low accuracy. Intraoperative Tg-FNA was reliable in detecting CNC metastases, although a higher cutoff is needed in this compartment compared to what has been reported for lateral lymph nodes. Lymphatic drainage and surgical manipulation might explain these findings. The careful interpretation of Tg-FNA in CNC should be adopted.

## Simple Summary

The early identification of metastatic lymph nodes in the central neck compartment (CNC) is crucial in tailoring the extent of the surgery in differentiated thyroid carcinoma. This prospective study explores the accuracy of intraoperative thyroglobulin measurement in the fine needle aspiration (Tg-FNA) of CNC lymph nodes to detect hidden metastases. We compared two methods: identifying lymph nodes using the blue dye technique versus the inspection and palpation of suspicious nodes. All lymph nodes selected were subjected to Tg-FNA and then histologically analyzed to identify metastases. Our findings revealed that the blue dye technique was unreliable. Intraoperative Tg-FNA without blue dye was reliable in detecting CNC metastases, although a higher Tg-FNA cutoff is needed in this compartment compared to what has been reported for lateral lymph nodes in the literature. Lymphatic drainage and surgical manipulation might explain these findings. With careful interpretation, intraoperative Tg-FNA can guide surgical decisions and avoid unnecessary CNC lymph node dissections.

## 1. Introduction

The appropriate management of occult cervical lymph node metastasis in differentiated thyroid carcinoma (DTC), mostly papillary thyroid carcinoma, is debated [1,2]. Occult regional lymph node involvement occurs in 25% to 90% of DTC and is associated with higher tumor recurrence rates; however, there is no evidence that the removal of these lymph node metastases confers a survival advantage. The central neck compartment (CNC), particularly the paratracheal and pretracheal regions, is the most common site for these metastases. Although DTC commonly spreads to regional lymph nodes, the prognostic value of this regional spread is still uncertain [3,4]. According to the AJCC 8th edition, the presence of CNC lymph node metastasis does not result in an upgrade in staging in patients younger than 55 years [5]. On the other hand, some authors have reported that lymph node metastasis is a significant prognostic factor that increases the risk of loco-regional recurrence and worsens survival [6,7,8]. However, routine prophylactic CNC dissection in patients with DTC is not yet accepted as a standard approach due to risks of hypocalcemia and recurrent laryngeal nerve injury [9,10].

The decision to perform central neck dissection during thyroidectomy typically depends on whether malignant lymph nodes can be identified preoperatively. When lymph node metastases in CNC are confirmed, the standard approach is to resect all affected lymph nodes. The preoperative detection of cervical lymph node metastases is currently performed with neck ultrasound (US) combined with cytology and the measurement of thyroglobulin (Tg) in the fine needle aspiration washout fluid (Tg-FNA) from the lymph node [11]. Although this technique is easily performed in lateral cervical lymph nodes, with very high diagnostic accuracy in detecting DTC metastasis, it has lower performance in CNC investigation. In fact, in several cases, US has a lower detection rate for metastatic lymph nodes in CNC, making it challenging to perform FNA [12,13]. Furthermore, the close proximity of these lymph nodes to the thyroid gland can lead to false-positive Tg-FNA results, due to the Tg contamination of the needle through the aspiration route [14].

The identification of the first CNC lymph nodes involved in DTC, defined as sentinel lymph nodes (SLN), has emerged as a potential technique for the early detection of metastases. The prompt identification or exclusion of SLN metastases could guide the appropriate surgical management of CNC lymph nodes. Several studies have explored different techniques for SLN detection [15,16,17]. In 1998, Kelemen et al. pioneered the vital blue dye technique for SLN biopsy in 17 thyroid cancer patients [15], while subsequent reports by Gallowitsch et al. and Rettenbacher et al. described the use of a radiotracer with intra-operative counting using a hand-held gamma probe [16,17]. In 2006, Pelizzo et al. evaluated the accuracy of SLN mapping via the intratumoral injection of blue dye in a large series of patients with papillary thyroid carcinoma (PTC). In 2008, they further investigated the efficacy of the ^99m^Tc nano-colloid SLN procedure in 99 PTC patients [18,19]. Despite these efforts, subsequent studies have not yielded conclusive results, and none of the above procedures has been validated for routine clinical use [20,21]. As a result, SLN identification is currently not part of the standard clinical practice in thyroid cancer surgery. Given these inconclusive results and the established high diagnostic accuracy of preoperative Tg-FNA in detecting lateral neck lymph nodes metastases, we aimed to compare the diagnostic performance of intraoperative Tg-FNA with or without the blue dye technique in identifying CNC metastases in a series of patients undergoing surgery for suspected DTC.

## 2. Materials and Methods

### 2.1. Study Design

We performed a prospective, single-center, non-profit, clinical study to evaluate the diagnostic sensitivity and specificity of intraoperative Tg-FNA and the blue dye technique compared to Tg-FNA alone in detecting CNC metastases in patients with suspected DTC.

### 2.2. Setting and Study Approval

The study was carried out at the Endocrinology Unit and Surgery Unit in the University Hospital of Cagliari. It was granted approval by the Institutional Review Board of the University Hospital of Cagliari. The ethical code of this study was PG/2017/3273-3.42/2022. All procedures were conducted in accordance with the Declaration of Helsinki, with the principles of good clinical practice, and with the GDPR regulations for privacy. Each patient provided written informed consent after a full explanation of the purpose and nature of all procedures used, ensuring ethical and transparent involvement in the research. Informed written consent was obtained from all subjects involved in the study.

### 2.3. Patients

Data analysis was conducted in 37 consecutive patients (28 female and 9 male) undergoing total thyroidectomy at Cagliari University Hospital for suspected DTC between November 2017 and November 2019. The sample size could not be predetermined, as no comparable studies were available in the existing literature to serve as a reference. All enrolled patients fulfilled the following inclusion criteria and exhibited none of the following exclusion criteria.

Inclusion criteria: (1) men and women aged 18–75 years; (2) suspected DTC, based on cytology results and clinical and ultrasound features; (3) written, signed informed consent, including compliance with requirements and restrictions listed in the consent form.

Exclusion criteria: (1) patients with a presurgical diagnosis of lymph node metastasis confirmed by FNA; (2) presence of distant metastases; (3) patients subjected to any previous neck surgery (e.g., thyroidectomy, lymph node dissection, or other cervical procedures); (4) mental illness preventing comprehensive informed consent.

### 2.4. Outcomes

The primary endpoint of the study was the assessment of the sensitivity and specificity of intraoperative Tg-FNA and the blue dye technique compared to Tg-FNA alone in identifying CNC metastases in patients with suspected DTC.

The secondary objective was to establish the optimal Tg-FNA cutoff for the detection of CNC metastases in patients with suspected DTC in intraoperative surgical settings.

### 2.5. Procedures

Preoperatively, all patients underwent blood tests to assess their serum Tg levels. The patients were then divided into two groups in alternating order. Group A included 15 patients (23 CNC lymph nodes) subjected to blue dye injection during surgery, while group B included 22 patients (35 CNC lymph nodes) who were not subjected to blue dye injection. Age and gender were equally distributed between the groups. The mean age was 48 years (standard deviation (SD) ±12) with 70.6% of females in group A, and it was 49 years (SD ±14) with 78.8% of females in group B. In order to identify suspicious lymph nodes, in the first 15 patients (group A), at the beginning of the surgery, the suspicious thyroid nodule was exposed by the lateralization of the strap muscles. Before the mobilization of the gland, 0.2 mL of patent blue dye was injected directly into the nodule with a 27-gauge needle. In the case of multiple suspicious nodules, the predominant nodule was injected. The lymph node that was stained in response was identified as suspicious. In the other 22 patients (group B), this procedure was not used, and suspicious lymph nodes were selected by inspection and palpation (size > 1 cm and/or hard consistency). Group B included more patients because, after the initial procedures, challenges in identifying suspicious lymph nodes using the blue dye technique became evident. Consequently, the last patients enrolled were all assigned to group B. All CNC lymph nodes identified by both the procedures were intraoperatively subjected to Tg-FNA. Intraoperative FNA was performed using 22- to 25-gauge needles attached to a 10 mL syringe, which was inserted into the lymph node and repeatedly moved within it until the needle hub was filled with material. The needle was then washed out with 1 mL of the diluent provided by the Tg assay kit, used to dilute the standard curve, and the solution was subsequently processed for Tg measurement. All lymph nodes submitted to aspiration were marked with suture tags to ensure proper association with the corresponding histology report. A total thyroidectomy was then performed, followed by the excision of the main lymph nodes in the ipsilateral compartment of the suspicious ones. After surgery, all histological lymph nodal samples were fixed in formalin and stained with hematoxylin and eosin. Definitive histological reports were performed by the same trained pathologist, who was blinded to the Tg-FNA values. Lymph nodes were classified as either metastatic or non-metastatic, with all metastatic cases showing macro-metastases; the Tg-FNA values were then compared with the corresponding histological findings.

Serum Tg and Tg-FNA values were assayed by a chemiluminescent assay (Immulite 2000 Thyroglobulin, Diagnostic Products Corporation, Los Angeles, CA, USA) with diagnostic sensitivity of 0.5 ng/mL.

### 2.6. Statistical Analysis

The assumption of normality was assessed using the Shapiro–Wilk test. Continuous variables were expressed as the median and interquartile range (IQR) for non-normally distributed data. Pairwise comparisons between groups were conducted using the independent Mann–Whitney U test. Correlations were studied with the Spearman’s rho test. The receiver operating characteristic (ROC) curve was adopted to identify the best cutoff value. The sensitivity and specificity of each group were then calculated by the Gale and Gambino formula. Graphical representations and statistical analyses were completed using the GraphPad Prism software (version 10.1.1). A *p* value of less than or equal to 0.05 was considered statistically significant for all tests.

## 3. Results

### 3.1. Comparison of Tg-FNA Values Between Metastatic and Non-Metastatic Lymph Nodes

**Figure 1:**
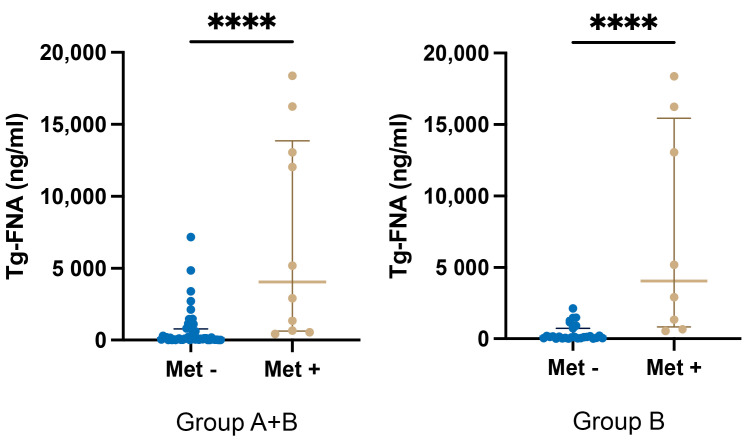
(**A**)—Comparison between non-metastatic and metastatic Tg-FNA values in groups A + B; (**B**)—comparison between non-metastatic and metastatic Tg-FNA values in group B; comparisons between groups were conducted using 2-tailed, unpaired Mann–Whitney tests. Tg-FNA, thyroglobulin fine needle aspiration; **** *p* < 0.0001.

Overall, metastatic lymph nodes showed significantly higher median Tg-FNA levels (4063 ng/mL, IQR 623–13,859) compared to non-metastatic ones (112 ng/mL, IQR 29.40–802; *p* < 0.0001) in both groups (Table 1 and Figure 1, panel A).

In group A, the blue dye diffused quickly and nonspecifically, staining multiple CNC lymph nodes, which made this technique unreliable. Although challenging, a total of 23 suspicious lymph nodes were identified; of these, two were metastatic and 21 were non-metastatic. The metastatic lymph nodes showed a median Tg-FNA level of 6236 ng/mL (IQR 430–12,041), while the non-metastatic ones had a median Tg-FNA level of 99.20 ng/mL (IQR 16.6–955). As expected, the Tg-FNA levels were higher in the metastatic group. However, the difference was not statistically significant due to the very limited size of the metastatic group (n = 2).

In group B, 35 clinically identified lymph nodes were examined. Among these, eight were metastatic and 27 were non-metastatic. Metastatic lymph nodes showed significantly higher median Tg-FNA levels (4063 ng/mL, IQR 822.8–15,446) compared to non-metastatic ones (121 ng/mL, IQR 43.1–725; *p* < 0.0001) (Table 1 and Figure 1, panel B).

There was no statistically significant difference between the Tg-FNA values of non-metastatic lymph nodes in the two groups. However, the non-metastatic lymph nodes in group A showed isolated cases of markedly elevated Tg-FNA values that exceeded the upper range limit observed in group B (2136 ng/mL). Specifically, four outliers in group A had values ranging between 2725 and 7168 ng/mL. Lastly, the comparison of the Tg-FNA values in the metastatic lymph nodes between group A and group B revealed no significant difference (*p* = 0.8633).

### 3.2. Identification of the Best Tg-FNA Cutoff and Accuracy Between Groups

**Figure 2:**
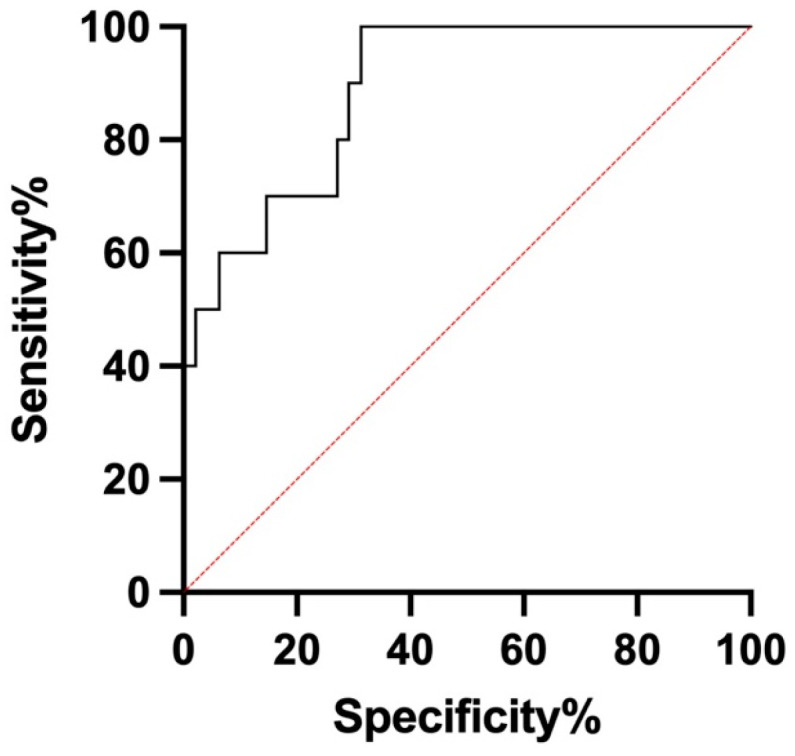
Receiver operating characteristic (ROC), AUC 0.8896, Std Error 0.04855, 95% CI 0.7944 to 0.9847, *p* value < 0.0001.

**Figure 3:**
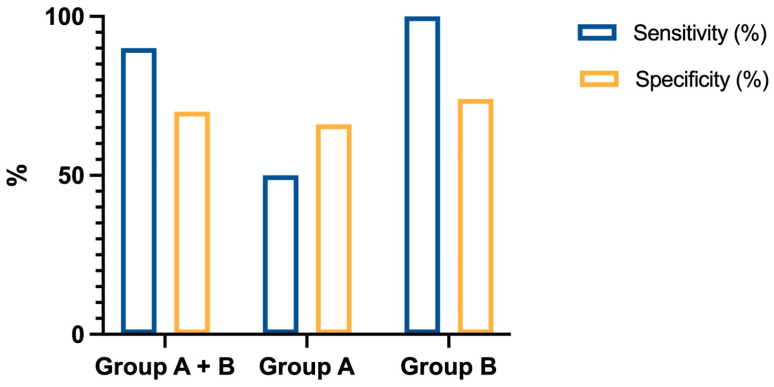
Sensitivity and specificity using the Tg-FNA cutoff of 500 ng/mL in group A + B, group A, and group B.

We performed a receiver operating characteristic (ROC) curve analysis to determine the optimal cutoff value that provided the best balance of sensitivity and specificity for the entire series. Additionally, we calculated the area under the curve (AUC) to assess the diagnostic performance of Tg-FNA. According to the ROC analysis, the best cutoff was 500 ng/mL. The AUC was 0.8896, confirming the good diagnostic accuracy (Figure 2). Among the metastatic lymph nodes from the entire series, almost all (9/10) had Tg-FNA > 500 ng/mL, while only one lymph node had a value < 500 ng/mL, resulting in sensitivity of 90% (Table 2). Among the benign lymph nodes, 34 had Tg-FNA < 500 ng/mL, and 14 had a value > 500 ng/mL, resulting in overall specificity of 70%. The positive predictive value (PPV) was 39% and the negative predictive value (NPV) was 97%. The 500 ng/mL cutoff showed the best overall diagnostic accuracy in group B, achieving 100% sensitivity, 74% specificity, a 53.3% PPV, a 100% NPV, and 80% accuracy. However, the diagnostic accuracy was lower in group A, with sensitivity of 50%, specificity of 66%, a PPV of 12.5%, an NPV of 93.3%, and accuracy of 65.2%. Notably, Tg-FNA alone had a higher PPV (53.3%) and NPV (97%) compared to Tg-FNA with the blue dye. These results are detailed in Table 2 and Figure 3.

### 3.3. Serum Tg Values Did Not Interfere with Tg-FNA Values

**Figure 4:**
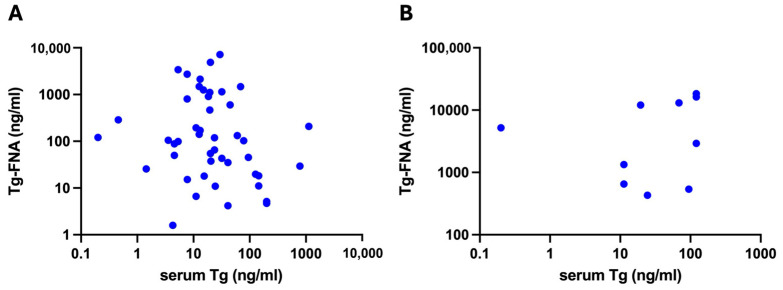
(**A**) Absence of correlation between serum Tg and Tg-FNA values in group A + B non-metastatic lymph nodes (r= −0.2201, 95% CI −0.4865 to 0.08374, *p=* 0.1416). (**B**) Absence of correlation between serum Tg and Tg-FNA values in group A + B metastatic lymph nodes (r= 0.3939, *p=* 0.2583). Log 10 scale was applied to both axes to improve visualization. Correlation was analyzed using non-parametric 2-tailed Spearman’s test. Tg-FNA, thyroglobulin fine needle aspiration; Tg, thyroglobulin.

Since serum Tg may potentially alter the Tg-FNA concentration due to hematic contamination, to exclude any interference, we compared the preoperative serum Tg values between the patients in group A and group B. The results showed no significant difference between the two groups: the median value in group A was 22 ng/mL (IQR 11.6–114), while, in group B, it was 9.50 ng/mL (IQR 5.3–41). Additionally, we analyzed the correlation between the serum Tg and Tg-FNA values. Spearman’s test showed the absence of a correlation between the serum Tg and Tg-FNA values, both in non-metastatic (r= −0.2201) and metastatic lymph nodes (r = 0.3939), confirming that there was no hematic contamination (Figure 4).

## 4. Discussion

The management of CNC lymphadenopathy in DTC remains a debated topic, with different approaches ranging from blind lymph node sampling and the selective removal of suspicious lymph nodes to radical CNC dissection. However, CNC lymph node dissection extends the surgical time and is associated with increased morbidity. The early identification of DTC patients with metastatic CNC lymph nodes could help to tailor the extent of surgery, minimizing unnecessary CNC dissections [22] while improving patients’ prognosis and follow-up. High-resolution cervical US is widely regarded as the most sensitive method of differentiating metastatic from benign lymph nodes, particularly in the lateral neck compartment. In newly diagnosed DTC patients, preoperative US often detects metastatic lymph nodes that might otherwise be missed on palpation [23]. However, a definitive diagnosis relies on cytology and Tg-FNA confirmation. Despite its utility, US has technical and anatomic limitations, making it less accurate in identifying lymph node metastases in the CNC—the neck compartment that is most frequently involved in DTC [24,25].

Several techniques have been proposed to achieve the prompt identification of lymph node metastases, including blue dye injection and lymphoscintigraphy with radioisotopes, sometimes used in combination. However, these methods have yielded inconclusive results [26,27,28]. Tg-FNA of clinically or sonographically suspicious lymph nodes is a widely accepted tool for the preoperative detection of metastasis in the lateral neck compartment. However, its role in CNC lymph node remains uncertain, primarily due to the lack of a well-defined cutoff value [29]. Moreover, this technique has not been thoroughly investigated in the intraoperative setting.

Building on this concept, our study aimed to compare the effectiveness of the intraoperative Tg-FNA of suspicious lymph nodes identified with blue dye versus the Tg-FNA of lymph nodes detected without blue dye in identifying metastatic CNC lymph nodes, and to establish a specific Tg-FNA cutoff for this compartment in intraoperative surgical settings.

The literature shows controversial results for the efficacy of blue dye [20,30]. In our study, the blue dye injection technique was strongly limited by the rapid diffusion of the dye into the surrounding tissues, making the precise identification of suspicious lymph nodes challenging. Similarly, lymphatic vessel damage during injection, the accidental staining of the surrounding tissues, and difficulties in tracking the dye outside the CNC were also reported by other authors [26]. Additionally, this false-positive staining may complicate preservation efforts regarding the parathyroid gland and the inferior laryngeal nerves [26].

The analysis of the Tg-FNA results of the lymph nodes identified by blue dye were separately evaluated and collected in group A, while those not subjected to colorant injection were classified into group B. As expected, the metastatic lymph nodes in both groups showed significantly higher median Tg-FNA levels compared to non-metastatic lymph nodes. However, we detected measurable and, in some cases, markedly elevated Tg-FNA levels in several non-metastatic lymph nodes. These elevated values could be attributed to the release of Tg from the thyroid gland into the lymphatic vessels. Evidence of Tg in the human lymphatic system has been reported [31]. Furthermore, studies in mice and monkeys have demonstrated that the Tg levels can increase in the thyroid lymph following exogenous TSH administration or the external massage of the gland [32]. This may explain our findings, as surgical manipulation likely triggered Tg’s transit into the lymphatic system. Additionally, the mechanical effect of blue dye injection could have further amplified this phenomenon, thereby justifying, at least in part, those strongly outlying Tg-FNA values observed in the non-metastatic lymph nodes of group A.

We then performed an ROC analysis on the entire series, demonstrating good accuracy (AUC 0.8896) and identifying 500 ng/mL as the optimal cutoff, offering the best balance between sensitivity and specificity. Notably, Tg-FNA guided exclusively by inspection and palpation (group B) exhibited higher sensitivity, specificity, PPV, and NPV compared to Tg-FNA performed on suspicious lymph nodes selected using the blue dye technique (group A). The lower accuracy of the cutoff in group A was probably due to the outlier values mentioned above. Overall, when combining group A and group B, Tg-FNA achieved sensitivity of 90% and specificity of 70%. The cutoff value that we propose is higher than those reported in the literature, likely due to the considerable heterogeneity across studies, which may influence the Tg-FNA results [29]. It should be underlined that the intraoperative surgical setting introduces additional variables, such as the degree of thyroid manipulation, which could differ based on individual patient features, disease extension, and the surgical approach. Moreover, most of the studies carried out in intraoperative settings were conducted in patients who had already undergone thyroidectomy and did not differentiate the Tg-FNA values from the lateral neck compartment and CNC lymph nodes [33]. Among the few intraoperative studies with thyroid in situ, D’Angeli S et al. identified a cutoff of 60 ng/mL, including both lateral and CNC compartment lymph nodes [34]. Similarly, Wang Y et al. proposed an overall cutoff of 146 ng/mL as an average from both the lateral neck compartment and CNC; however, this cutoff showed lower diagnostic accuracy in the CNC, suggesting the need for a higher threshold for this compartment [35]. Conversely, our study was specifically designed to intraoperatively evaluate CNC lymph nodes in patients with thyroid in situ. Compared to the other intraoperative studies, our findings provide clear evidence that the Tg-FNA cutoff for metastatic CNC lymph nodes is higher.

We also investigated whether the serum Tg levels interfered with Tg-FNA evaluation and found no correlation between the two parameters. This is consistent with our previous preoperative study, which similarly demonstrated the absence of serum Tg interference [36]. These results support the assumption that the elevated intraoperative Tg-FNA levels observed also in non-metastatic CNC lymph nodes are attributable to lymphatic rather than hematic contamination. Tg seems to be inherently present in CNC lymph nodes due to normal lymphatic drainage from the thyroid gland, and its level can be further raised by surgical manipulation, blue dye injection, or even increased Tg turnover. This finding highlights the need for the careful interpretation of the Tg-FNA results in CNC lymph nodes, particularly intraoperatively, given the physiological transit of Tg in this compartment, amplified by surgical thyroid manipulation. To minimize this effect, the surgical handling of the thyroid gland should be limited to facilitate the exposure of CNC lymph nodes. This concept could be extended to the presurgical evaluation of CNC lymph nodes, which should adopt similar measures, such as performing the FNA of the lymph node before the thyroid nodule.

Overall, due to its high NPV, intraoperative Tg-FNA is demonstrated to be a promising tool that could become a routine part of clinical practice in guiding surgical decisions and reducing unnecessary CNC lymph node dissections. The first step of the procedure would be the surgical exposure of CNC, followed by the inspection and palpation of the lymph nodes to identify suspicious ones, which would then be subjected to Tg-FNA. Tg measurement should be processed in a nearby laboratory, with the results available within 30 to 40 min. This time would allow the surgical team to proceed with the thyroidectomy while awaiting the Tg-FNA results. If positive, CNC dissection could be performed in the same surgical session. This approach could be particularly useful in patients with Bethesda V cytology. It offers a rapid, cost-effective, and reproducible solution. However, further studies are required to confirm its feasibility and reproducibility across various clinical contexts.

Finally, we are aware that one of the main limitations of this study is the relatively small sample size, with only 37 patients and 55 lymph nodes evaluated. Another limitation is the single-center nature of our study, which restricted the number of enrolled patients and limited the generalizability of the results. On the other hand, this single-center setup ensured procedural consistency, as the same surgical team conducted all procedures, thereby minimizing variability. Nonetheless, factors such as the degree of surgical manipulation of the thyroid gland and lymph nodes were not fully standardized. Reducing manipulation during future procedures could further improve the accuracy and reliability, minimizing Tg release from the thyroid and allowing the identification of a more accurate Tg-FNA cutoff for CNC metastatic lymph nodes. To definitively answer these questions, randomized controlled trials involving a larger cohort of patients are needed, possibly including patients with different thyroid diseases.

## 5. Conclusions

Our study demonstrated the low accuracy of blue dye injection in correctly identifying CNC lymph node metastases, as well as its potential to interfere with the Tg-FNA values. In contrast, the intraoperative Tg-FNA of CNC lymph nodes proved to be a reliable tool for the detection of DTC metastases, even though a higher cutoff appears to be necessary compared to what is reported in the literature for the lateral compartment. Specifically, the Tg-FNA cutoff of 500 ng/mL showed promising sensitivity with a high NPV in identifying CNC lymph node metastases. Given the lymphatic Tg diffusion observed in CNC lymph nodes, which is amplified by thyroid manipulation, careful interpretation of the Tg-FNA results should be adopted. To further refine the diagnostic accuracy of intraoperative Tg-FNA for CNC lymph nodes, reducing surgical manipulation, standardizing the procedures, and expanding the patient cohort should be considered in larger future studies. These efforts could provide a clearer understanding of the optimal cutoff values and improve the overall reliability of this approach.

## Figures and Tables

**Table 1:** Distribution of median Tg-FNA lymph node values in group A and group B.

		N Lymph Nodes (tot = 58)	Tg-FNA Median (IQR)
Group A Blue + (n = 23)	Met −	21	99.20 ng/mL (16.6–955 ng/mL)
	Met +	2	6236 ng/mL (430–12,041 ng/mL)
Group B Blue − (n = 35)	Met −	27	121 ng/mL (43.1–725 ng/mL)
	Met +	8	4063 ng/mL (822.8–15,446 ng/mL)

Met, metastatic; Tg-FNA, thyroglobulin fine needle aspiration; IQR, interquartile range.

**Table 2:** Distribution of lymph nodes and definitive histology using the 500 ng/mL cutoff.

Group A (n = 23)	**Tg-FNA > 500 ng/mL** **(n = 8)**		**Tg-FNA < 500 ng/mL** **(n = 15)**	
	Met – n = 7	Met + n = 1	Met – n = 14	Met + n = 1
Group B (n = 35)	**Tg-FNA > cutoff (500 ng/mL)** **(n = 15)**		**Tg-FNA < cutoff (500 ng/mL)** **(n = 20)**	
	Met – n = 7	Met + n = 8	Met – n = 20	Met + n = 0

Met, metastatic; Tg-FNA, thyroglobulin fine needle aspiration.

## Data Availability

The original contributions presented in this study are included in the article. Further inquiries can be directed to the corresponding author.
